# Author Correction: Progressive plasticity during colorectal cancer metastasis

**DOI:** 10.1038/s41586-024-08560-0

**Published:** 2025-01-09

**Authors:** Andrew Moorman, Elizabeth K. Benitez, Francesco Cambuli, Qingwen Jiang, Ahmed Mahmoud, Melissa Lumish, Saskia Hartner, Sasha Balkaran, Jonathan Bermeo, Simran Asawa, Canan Firat, Asha Saxena, Fan Wu, Anisha Luthra, Cassandra Burdziak, Yubin Xie, Valeria Sgambati, Kathleen Luckett, Yanyun Li, Zhifan Yi, Ignas Masilionis, Kevin Soares, Emmanouil Pappou, Rona Yaeger, T. Peter Kingham, William Jarnagin, Philip B. Paty, Martin R. Weiser, Linas Mazutis, Michael D’Angelica, Jinru Shia, Julio Garcia-Aguilar, Tal Nawy, Travis J. Hollmann, Ronan Chaligné, Francisco Sanchez-Vega, Roshan Sharma, Dana Pe’er, Karuna Ganesh

**Affiliations:** 1 Computational and Systems Biology Program, Memorial Sloan Kettering Cancer Center, New York, NY USA; 2 Molecular Pharmacology Program, Memorial Sloan Kettering Cancer Center, New York, NY USA; 3 Weill Cornell/Rockefeller/Sloan Kettering Tri-Institutional MD-PhD Program, New York, NY USA; 4 Pharmacology Program, Weill Cornell Graduate School, New York, NY USA; 5 Department of Medicine, Memorial Sloan Kettering Cancer Center, New York, NY USA; 6 Department of Pathology and Laboratory Medicine, Memorial Sloan Kettering Cancer Center, New York, NY USA; 7 Department of Surgery, Memorial Sloan Kettering Cancer Center, New York, NY USA; 8 Tri-Institutional PhD Program in Computational Biology and Medicine, New York, NY USA; 9 Howard Hughes Medical Institute, Chevy Chase, MD USA; 10 Present Address: New York Genome Center, New York, NY USA; 11 Present Address: Case Western Reserve University, Cleveland, OH USA; 12 Present Address: Bristol Myers Squibb, Princeton, NJ USA

**Keywords:** Colorectal cancer, Tumour heterogeneity, Metastasis, Experimental models of disease, Metastasis

Correction to: *Nature* 10.1038/s41586-024-08150-0 Published online 30 October 2024

In the version of this article initially published, initials were used instead of the authors’ given names, and the second affiliation shown for Emmanouil Pappou (Department of Surgery, Memorial Sloan Kettering Cancer Center, New York, NY, USA) was incorrect. The names and affiliation have been amended in the HTML and PDF versions of the article. Additionally, the open-access licence has been updated from a Creative Commons Attribution-NonCommercial-NoDerivatives 4.0 International licence to a Creative Commons Attribution 4.0 International licence.

